# Low agreement between modified-Schwartz and CKD-EPI eGFR in young adults: a retrospective longitudinal cohort study

**DOI:** 10.1186/s12882-018-0995-1

**Published:** 2018-08-06

**Authors:** Michael Webster-Clark, Byron Jaeger, Yi Zhong, Guido Filler, Ana Alvarez-Elias, Nora Franceschini, Maria E. Díaz-González de Ferris

**Affiliations:** 1 Gillings School of Global Public Health, Chapel Hill, NC USA; 2 Departments of Pediatrics and Economics, University of North Carolina at Chapel Hill, Chapel Hill, NC USA; 3 Department of Biostatistics, University of Alabama at Birmingham, Birmingham, AL USA; 4 Departments of Paediatrics, Medicine, and Pathology & Laboratory Medicine, University of Western Ontario, London, ON Canada; 5 Hospital Infantil de México “Federico Gómez”, Universidad Nacional Autonóma de México, Mexico City, D.F Mexico; 6 Lawson Health Research Institute, and Children’s Hospital, London Health Sciences Centre, University of Western Ontario, 800 Commissioner’s Road East, Rm B1-135, London, ON N6A 5W9 Canada

**Keywords:** CKD, eGFR, CKD-EPI, Schwartz formula, Paediatric to adult transition

## Abstract

**Background:**

While there is a great deal of research updating methods for estimating renal function, many of these methods are being developed in either adults with CKD or younger children. Currently, there is limited understanding of the agreement between the modified new bedside Schwartz estimated glomerular filtration rate (eGFR) formula and the adult CKD-EPI formula in adolescents and young adults (AYAs) with chronic kidney disease (CKD) measured longitudinally.

**Methods:**

Longitudinal cohort study of 242 patients (10–30 years) with CKD, followed retrospectively in a single tertiary centre as they transitioned from the paediatric- to adult-focused settings. The study population came from a longitudinal cohort of AYAs undergoing healthcare transition at the STARx Program at the University of North Carolina, in the South-Eastern USA, from 2006 to 2015. We calculated and compared the eGFR using the new bedside Schwartz formula and the CKD-EPI eGFR. Measurements were repeated for each age in years. Agreement was tested using Bland & Altman analysis. Subgroup analysis was performed using the following age groups 10–15, 15–20, 20–25 and 25–30 years, glomerular and non-glomerular causes of CKD and height z-score.

**Results:**

Using repeated measures, concordance between the new Schwartz and CKD-EPI eGFR was low at 0.74 (95% C.I. 0.67, 0.79) at the lowest age range of 10–15, 0.78 (95% C.I. 0.71, 0.84) at age 15–20, 0.80 (0.70, 0.87) at ages 20–25, and 0.82 (95% C.I. 0.70, 0.90) at age 25–30. Discordance was worse in males and largest in the 10–15 year-old age group, and in patients with stunted growth.

**Conclusions:**

The Schwartz and CKD-EPI equations exhibit poor agreement in patients before and during the transition period with CKD-EPI consistently yielding higher eGFRs, especially in males. Further studies are required to determine the appropriate age for switching to the CKD-EPI equation after age 18.

**Electronic supplementary material:**

The online version of this article (10.1186/s12882-018-0995-1) contains supplementary material, which is available to authorized users.

## Background

Chronic kidney disease (CKD) is associated with significant morbidity in adolescents and young adults (AYAs), but the true prevalence of CKD in these patients is unknown [1]. What is known is that AYAs with end-stage kidney disease (ESKD) - those who need dialysis or a transplant - are less than 5% of all ESKD patients in the USA; yet, they have a 10-year survival of 70–85%; [2–4] CKD is a progressive disease which requires careful monitoring of kidney function. The estimated glomerular filtration rate (eGFR) is the most widely used surrogate marker of kidney function [5] and is typically calculated based on endogenous biomarkers such as serum creatinine or cystatin C [6]. In children, the new bedside Schwartz formula (developed in a USA cohort of children with CKD and subsequently referred to simply as the new or modified Schwartz formula) [7] is recommended; whereas in adults, the CKD-EPI formula based on cystatin C and serum creatinine (developed from large studies from different parts of the world and differing measured GFR methods) is endorsed through international guidelines [8]. These equations estimate renal function based upon various factors that may include: age, sex, height, and ethnicity. Both the adult and paediatric formulae advocate for the bivariate combination of cystatin C (measured against the new international reference materials [9]) and calibrated (isotope dilution mass spectrometry (IDMS) traceable) serum creatinine [10]. However, most centres use creatinine- based formulae only. For paediatric CKD patients, the new Schwartz formula is recommended, whereas the CKD-EPI formula is recommended by the Kidney Disease Outcomes Quality Initiative (KDOQI) guidelines for all adults while the best age cut-off is not well defined.

Most of the adult formulae such as the Cockcroft Gault, [11] the CKD-EPI formula [12] or the new CKD-EPI based on beta-trace protein and beta-2-microglobulin [13] are not suitable for paediatric patients. Selistre [12] advocates for the use of the new Schwartz formula in young adults, but this recommendation has not yet been validated. Our objective was to explore the extent of the agreement between the new Schwartz and the new CKD-EPI equations in a longitudinal, racially diverse cohort of AYAs with CKD. We hypothesized that the equations would become more similar as patients aged.

## Methods

### Study design

Descriptive retrospective longitudinal observational cohort study.

### Setting

Patients were recruited from the University of North Carolina *STAR*_*x*_ (Self-management and Transition to Adult-focused healthcare with R_x_ = treatment) Program (recruitment details noted in Ferris et al. 2015) [14–16].

### Participants

Patients from the *STAR*_*x*_ Program were eligible for inclusion in this study if they were prevalent or incident CKD cases followed annually at either the paediatric- or adult-focused nephrology clinics. Patients were excluded if they had spina bifida or muscular dystrophy, conditions that would drastically alter muscle mass. We consented patients and parents to access their electronic health record (system available since 1984) under a protocol and using forms approved by the University of North Carolina at Chapel Hill. The study adhered to the Declaration of Helsinki. The patients’ longitudinal medical and laboratory information since their first visit to the medical centre was obtained. For this study, we collected the patients’ basic demographic information (e.g., age, sex, and race), serum creatinine, anthropometrics (e.g., height and weight), and if applicable, dates of transplants and dialysis. Individuals were censored from the population after their first dialysis or after renal transplant due to potential inaccuracy in creatinine clearance estimation because of renal replacement therapy or graft function. Analyses (with the exception of the graphical population curves) required complete case data on age, race, sex, gender, and creatinine.

### Variables

The primary outcome variable was their eGFR, which was calculated by the new Schwartz or new CKD-EPI estimating equations based upon creatinine, with all required covariates abstracted from the medical record. These equations were chosen because cystatin C measurements were rarely, if ever, ordered during the time period covered by the study. Schwartz eGFRs were calculated using the new Schwartz estimating equation: [7].

$$ eGFR=0.413\ast \frac{Heigh{t}_{cm}}{Sc{r}_{\frac{mg}{dL}}}\  or\ eGFR=36.2\ast \frac{Heigh{t}_{cm}}{Sc{r}_{\frac{umol}{L.}}} $$

CKD-EPI eGFRs were estimated with the 2009 iterations of the CKD-EPI equation: [8]

$$ eGFR=C\ast {\left(\frac{SCr_{\frac{mg}{dL}}}{S}\right)}^k\ast \left({0.993}^{Age}\right)\ast \left(1+0.159\ast African\ American\right) $$

Where C = 144 and S = 0.7 if the patient was female and C = 141 and S = 0.9 if the patient was male. We used k = − 0.329 if the patient was female with serum creatinine ≤0.7, k = − 1.209 if the patient was female with serum creatinine > 0.7, k = − 0.411 if the patient was male with serum creatinine ≤0.9 and k = − 1.209 if the patient was male with serum creatinine > 0.9. For use with SI units, S and serum creatinine bounds were replaced with 61.9 for females and 79.6 for males; while the other coefficients remained the same.

### Measurement

The UNC laboratory measured creatinine using the OrthoClinical Diagnostics Vitros Creatinine slide. They used Jaffe’s reaction and switched to an enzymatic IDMS-traceable assay in May 2008; prior measurements were corrected by multiplying them by a factor of 0.95. All heights were measured with a standing stadiometer at routine clinic visits. Heights were cleaned to remove implausible values (i.e. > 300 cm) or values that were entered with incorrect units (i.e. a patient going from 100 cm to 254 cm back to 100 cm), and if a particular creatinine did not have a height associated with the visit, heights were interpolated as a weighted average of the previous and next height measurements. Measurements for each patient within each half-year were averaged to obtain an overall estimate for that half-year of age.

### Quantitative variables

Age was used as a continuous variable. Z-score of height was also used to divide patients into thirds relative to the z-score distribution of the overall cohort and allow assessment of concordance differences based upon relative stunted growth. Height z-scores were based on the Centers for Disease Control and Prevention’s growth charts [17].

### Bias

No formal methods were used to correct for systematic biases. However, patients were enrolled consecutively during the study period with a response rate of 97% and lack of time was the sole reason given for non-enrolment. Thus, the patient sample is representative of the patients UNC serves in terms of race, sex and insurance. Given that more than half of the individuals studied had an eGFR> 60 at study entry, there remains the possibility that the results could be biased due to systematic underestimation of the eGFR in that group using the Schwartz equation. However, bias of the Schwartz equation in children is greater in children with low GFR, rather than GFR close to normal [18].

### Statistical methods

We used graphical and statistical methods to capture changes in the extent of disagreement between the new Schwartz and the CKDEPI eGFR estimations as patients in our cohort aged. Locally weighted polynomial regression (LOESS) curves were generated for each measure of eGFR over time using R packages “ggplot2” [19] and “ggExtra” [20]. These curves represent the average of the eGFR from a given equation at each age across the total population (with each individual representing at most one data point at a given age). The closer the two lines are to one another at a given age, the more similar the overall population estimates generated by the new Schwartz and CKDEPI are. Similar graphs were constructed to compare the new Schwartz and CKDEPI eGFR estimation in relevant subgroups of interest with stratification by sex, race, type of chronic kidney disease, and z-score of height at current age.

Considering that we lack access to true measured GFR data in this longitudinal data set (i.e. inulin clearance), statistical disagreement was captured using the concordance correlation coefficient. This method assesses the extent of agreement for two measures of the same base quantity more effectively than a t-test or normal correlation coefficient [21]. A variant of this method has been adapted for use through repeated measurements on individuals, which allows us to leverage the full breadth of our longitudinal data set rather than relying on cross-sectional cuts. This method also allows us to estimate the concordance correlation coefficient across different age periods, assessing changes in our estimate of the concordance correlation coefficient as patients aged. We estimated concordance correlation coefficients using R package “cccrm” [22] and creatinine measurements from age 10–15, 15–20, 20–25, and 25–30; these age groups were chosen to make sure early adulthood could be split into two phases, as well as give one final category to assess agreement once individuals were far from adolescence. We also created a Bland-Altman plot (created using R package “BlandAltmanLeh” [23]) for each age group to visualize information about disagreement; unfortunately, unlike the concordance correlation coefficient, these plots cannot integrate and adjust for information about repeated measurements. To assess whether this would dramatically alter results, we created a second set of supplementary Bland-Altman plots in which individuals only contributed one dot per age group based upon their mean values in that age range. To assess whether subgroup effects might differ, sub-analyses were conducted across relevant strata of interest, including sex, race (African-American vs. not African-American), type of kidney disease (glomerular vs. not glomerular), and Z-score of height based upon the CDC’s growth charts [17].

To investigate potential differences in CKD stage categorization between the equations at age 18 differentially across height groups, we constructed an additional graph. Data management and table generation were performed in both SAS 9.4 for Windows (SAS Institute Cary, North Carolina) and R using packages “dplyr” [24] and “xtable [25].”

We used novel methods to estimate the proportions of the variance in the ratio of the CKD-EPI value to the new Schwartz value explained by height Z-score, type of CKD, age, sex, and race. The classical R^2^ for linear models measures the proportion of variance explained by a set of fixed predictors. Additionally, semi-partial R^2^ statistics measure the relative explanatory power of one specific predictor in the set after accounting for the others. Several analogues of the R^2^ are available for linear mixed models (LMMs), which are frequently used to characterize longitudinal data. Using the “lme4” [26] package, we fitted a LMM with the ratio of eGFRs modelled as a function of race, sex, age, and height Z-score. Using the “r2glmm” [27] package, measures of generalized explained variance were computed for the model and each predictor. Generalized explained variance may be interpreted as the proportion of multi-dimensional scatter that can be attributed to structural components (i.e. predictors) of a LMM [28].

### Missing data and loss to follow-up

We conducted a complete case analysis; that is, observations from a patient were only eligible for inclusion in our assessments if they were not missing data in any of the variables used to assess their eGFR. Once patients were lost to follow-up in creatinine measures, they no longer contributed to our eGFR comparisons.

## Results

### Participants

We enrolled 292 AYAs from the *STAR*_*x*_ Program who had the diagnosis of CKD. We excluded 50 AYAs from the analysis since they did not receive care at the clinic prior to their transplant or initiation of dialysis, yielding no usable creatinine measurements. No AYAs were identified with either spina bifida or neuromuscular dystrophy.

### Descriptive data

A majority (59%) of these AYAs had glomerular disease, and slightly less than half (47%) of patients had CKD stages ≥3 based upon an estimate of their renal function using the new Schwartz and the KDIGO categories [29] at their first recorded height. Over half (55%) of patients were male, and over one-third (38%) were African-American, representative of the population we serve. The median age at entry into this cohort was 13.6 years (interquartile range 11.4; 16.3 years) and the median duration of follow-up was 4.0 years (interquartile range 1.4; 6.3 years); both were not normally distributed. In general, AYAs had greater than average BMI, though there was a wide variance and while 26 AYAs had missing heights at all their visits these patients represented very little potential follow-up time overall. Otherwise, missing data for key variables was negligible. For full details of the descriptive population statistics, see Table 1.

**Table 1:** Population Description and Anthropometric Data

Variable	Mean (SD) or n (%)	Missing values n
Type of Kidney Disease		0
Glomerular	151 (62)	
Non-Glomerular	91 (38)	
Stage of Kidney Disease at first height value (by Schwartz)		26^a^
Stage 1	49 (23)	
Stage 2	65 (30)	
Stage 3	63 (29)	
Stage 4	24 (11)	
Stage 5	15 (7)	
Gender		0
Male	134 (55)	
Female	108 (45)	
Race		2
African-American	93 (38)	
Non-African American	147 (62)	
Age at cohort entry (years)		0
Mean (SD)	14.6 (4.4)	
Median (IQR)	13.6 (11.4, 16.3)	
Follow-up duration (years)		11^b^
Mean (SD)	4.5 (3.3)	
Median (IQR)	4.0 (1.4, 6.3)	
BMI at first possible calculation		9
Z-score if < age 18 (*n* = 207)	0.91 (1.14)	
BMI if > = age 18 (*n* = 36)	26.1 (6.7)	
Height		9
Z-score (at first height)	0.08 (1.27)	
Total eGFR measures across all patients	4094	
Age 10–15	1702 (41.6)	
Age 15–20	1715 (41.9)	
Age 20–25	715 (17.5)	
Age 25–30	549 (13.4)	

^a^These patients contributed only to the smoothed population curve for CKDEPI

^b^Generally patients contributing 1 measurement to the data set

### Main results

**Fig. 1:**
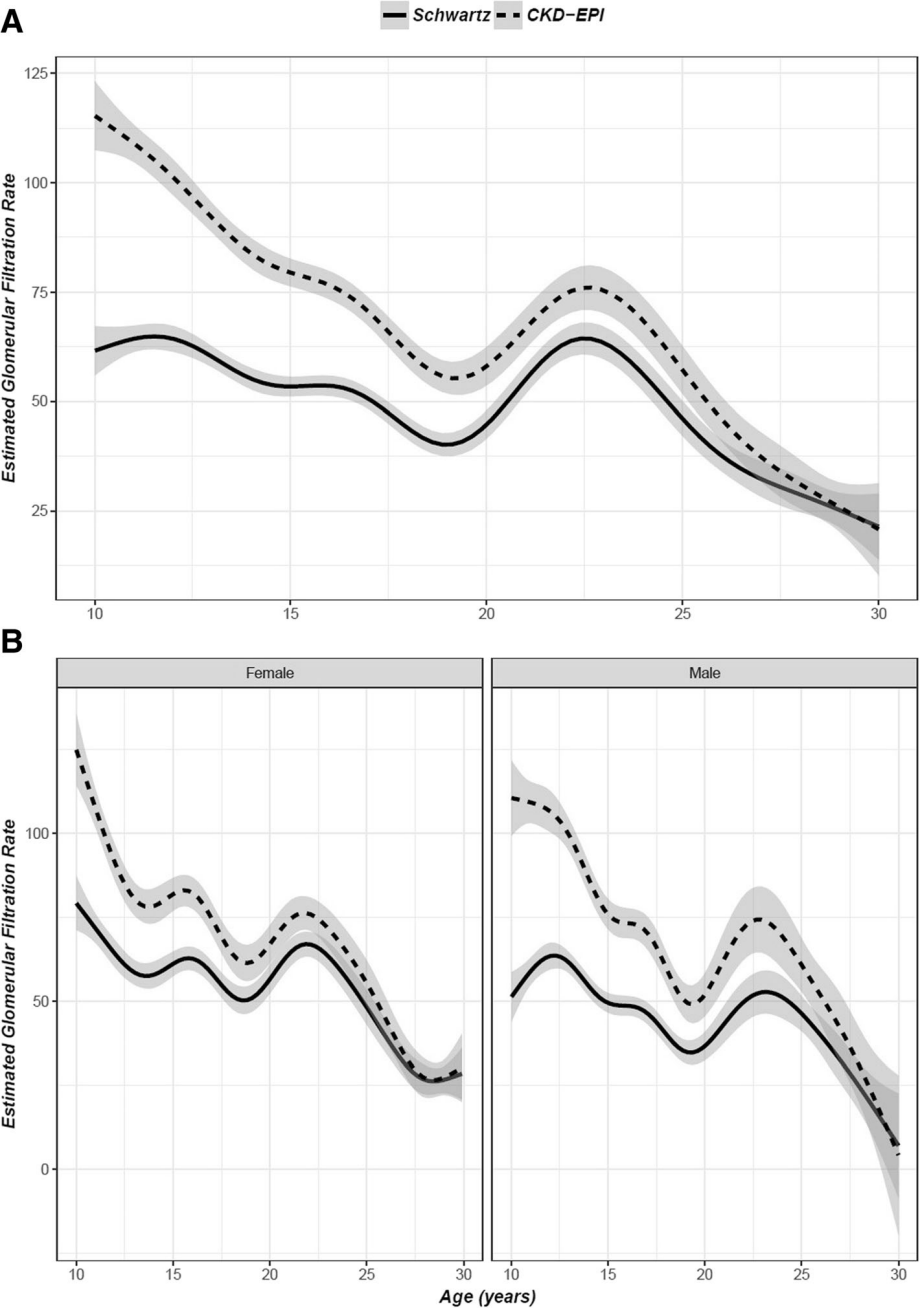
Estimated Glomerular Filtration Rates by Age and Estimating Equation. Legend: These figures describe trends in eGFR over time based upon the CKD-EPI equation (dashed line) or the Schwartz equation (solid line). Panel **a**) describes the overall population trajectory, while Panel **b**) depicts separate trajectories for males (on the right) and females (on the left)

Figure 1 is the smoothed population eGFR trajectories for the patients included in the study population across time. Panel A is the trajectory for the overall population, while Panel B shows stratified trajectories of men and women. This figure depicts agreement between equations improving over time as the trend lines become closer together with much more improvement in women in men. Additional file 1 shows additional smoothed population eGFR trajectories in additional subpopulations.

**Fig. 2:**
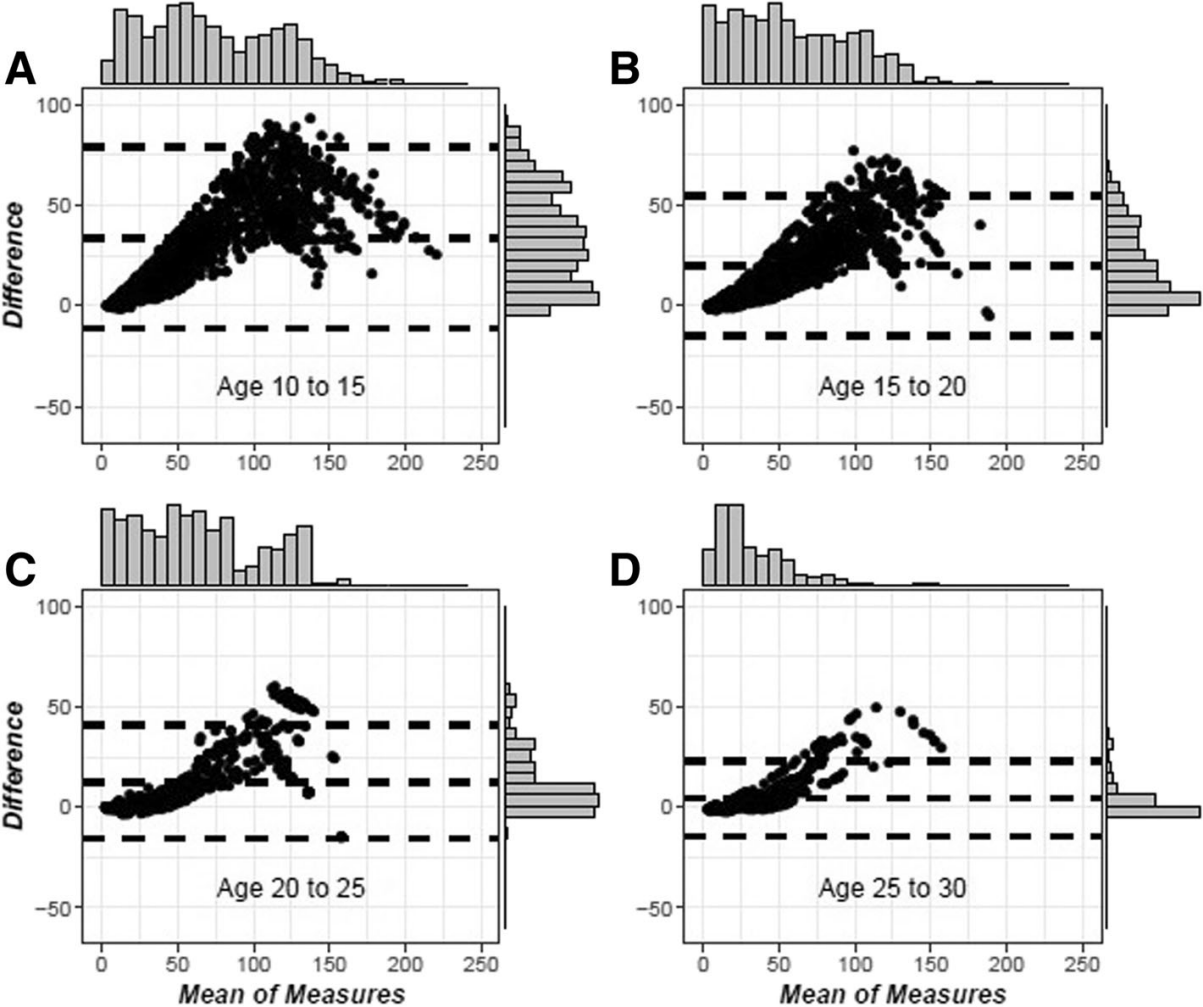
Bland-Altman Plots for Each of Four Age Groups. Legend: These Bland-Altman plots depict the agreement between our two measurements at each creatinine measurement within age intervals, with each panel from **a**) to **d**) representing a separate age group. The closer the central dashed line representing the mean to 0 within each plot, the better the agreement within that age group

**Fig. 3:**
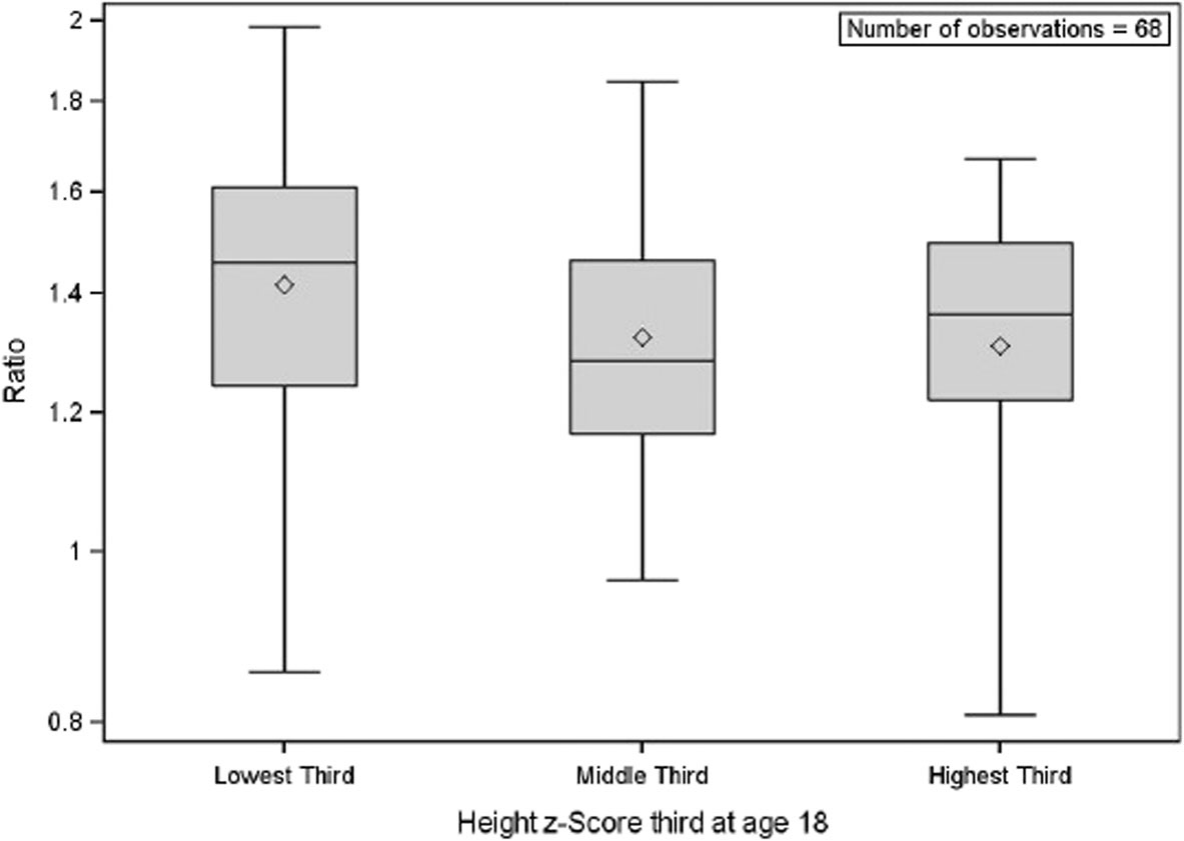
Ratio in Estimated Glomerular Filtration Rates (CKD-EPI/Schwartz) by Height Category at Age 18. Legend: These box and whiskers plot illustrate the value of the GFRs estimated by the CKD-EPI equation divided by the GFRs estimated by the Schwartz equation, with the diamonds representing means, the middle lines representing medians, the edges of the boxes representing quartiles, and the whiskers representing the range. The closer each value to 1, the more similar the equation estimates are for a given height category

Figure 2 is a panelled Bland-Altman plot for each of the four age categories. Additional file 2 contains a panelled Bland-Altman plot removing the potential for repeat observations. Both show improvement in agreement across the age groups. When the analysis with repeated measures was performed, concordance is initially low at 0.74 (95% C.I. 0.67, 0.79) at the lowest age range of 10–15 but trends upwards at 0.78 (95% C.I. 0.71, 0.84) at age 15–20, 0.80 (0.70, 0.87) at ages 20–25, and 0.82 (95% C.I. 0.70, 0.90) at age 25–30. This trend across age holds within every stratum. There is a markedly higher concordance in every age range comparing females to males. Females have a value of 0.82 (95% C.I. 0.73, 0.88) at age 10–15 compared to males’ 0.69 (95% C.I. 0.59, 0.77) at age 10–15, 0.88 (95% C.I. 0.80, 0.93) compared to 0.73 (95% C.I. 0.62, 0.82) at age 15–20, 0.89 (95% C.I. 0.79, 0.94) compared to 0.76 (95% C.I. 0.59, 0.86) at age 20–25, and 0.92 (95% C.I. 0.83, 0.96) compared to 0.76 (95% C.I. 0.52, 0.89) at age 25–30, but none of the other stratification categories showed a large difference that was sustained across all age categories. For a full list of stratum-specific concordance correlation coefficient estimates including gender, race, glomerular versus non-glomerular disease and height z-score, see Additional file 3. Figure 3 presents a chart assessing the ratio between the CKD-EPI and Schwartz equations among patients at age 18 with patients divided into categories by Z-score. There is a trend towards a larger ratio (greater disparity) in stunted patients.

Table 2 summarizes the semi-partial R^2^ estimates for specific predictors from the overall model with R^2^ = 0.9 with approximate 95% confidence limits. Age explained most of the variance, followed by sex, then race, then height Z-score, and finally followed by type of CKD.

**Table 2:** Semi-partial R^2^ Estimates for Each Predictor

Ranking	Variable	Rsq	Upper 95% CL	Lower 95% CL
1	Age	0.8105	0.8187	0.8022
2	Gender	0.5186	0.5373	0.4998
3	Race	0.3643	0.3861	0.3425
4	Height z-score	0.1107	0.1293	0.09319
5	Type of CKD	0.001986	0.00576	0.0001739

## Discussion

### Key results

We explored the extent of agreement between the new Schwartz and the CKD-EPI equations for eGFR estimation in a longitudinal, racially diverse cohort of AYAs with CKD, with the intent to provide clinical recommendations for the use of these equations in patients with CKD transitioning into adulthood. We found disagreement between the Schwartz and CKD-EPI equations at all ages in this CKD cohort. The disagreement was largest in patients aged 10–15 years old, but there was better agreement as patients aged. CKD-EPI consistently estimated higher GFRs compared to the Schwartz measurements across all age groups and observed strata. In the absence of a gold standard GFR measurement, we cannot state which formula is better. Disagreement between the CKD-EPI [8] and Schwartz [7] equations was lower among female than male patients at every age, perhaps due to the lower muscle mass or less pronounced stunted growth in women. Our analyses also exhibited that age and sex are both important components of the overall variance between the two renal function estimating equations, reinforcing that both attributes play important roles in the relative performance of the two equations. The use of a sex-specific adjustment in CKD-EPI may have contributed to the fact that the equation performed more comparably to Schwartz in women than in men, though this bears further investigation. Most importantly, the switch from Schwartz to CKD-EPI eGFR estimation introduced discontinuity across our entire analysis due to discordance between both formulae.

### Limitations

We do not have a gold-standard measured GFR and can thus only assess agreement rather than which formula is most accurate. However, CKD-EPI and the new Schwartz [7] eGFR formulae have been well validated in their respective age groups. Due to the data’s abstraction from electronic medical health records, there may be concerns about the accuracy or reliability of these lab measures. There are also concerns with missing data on height preventing estimation of GFRs using the Schwartz equations for some of the patients at some time points, but we overcame this obstacle by imputing height values between measurements. While this is a limitation, growth is pretty linear, and the error due to height imputation can be considered negligible. Another limitation is due to the higher number of measurements in the younger age groups (see Table 1).

### Interpretation

Overall, our study demonstrates that among AYAs with CKD, the CKD-EPI and Schwartz equations are discordant during the transition-age period and early adulthood. Selistre et al. [12] examined the difference in accuracy of the two equations across a broad spectrum of age categories using cross-sectional broad categories of age (2–12, 13–17, 18–40, 41–64, > = 65 years-old) but did not focus on the paediatric-adult transition. In Selistre’s study, a gold standard GFR measurement was performed, and he concluded that the Schwartz formula might be more accurate for young adults. Other studies assessing potential age cut-offs for kidney function equation [30] switching used measured GFRs for comparison or formulae incorporating both cystatin C and serum creatinine [31]. These studies identified similar trends in agreement as patients aged. A very recent study by Ng et al. compared creatinine and creatinine and cystatin C-based eGFR formulae in young adults with the diagnosis of paediatric CKD aged 18–26 years of age and concluded “clinicians should be aware that individually the paediatric and adult serum creatinine-based estimates of GFR had large discrepancies among emerging adults with paediatric CKD.” The group recommends taking the average of paediatric and adult serum creatinine-based formulae (i.e. the new Schwartz bedside and the CKD-EPI creatinine only formulae) as a valid tool for clinical use [32]. Of course, this approach needs further validation, but the results by Ng et al. are fully aligned with our findings. The current contribution augments the findings and suggests that this approach may be needed until age 30.

### Generalizability

The results of this study apply to other nephrology centres and possibly other diseases where eGFR is an important biomarker in similar settings within North America and other populations with a mix of Caucasian, African American and Hispanic patients. Groups with different ethnic makeup may have differing agreement between the two equations, although our study did not show this.

## Conclusion

The new Schwartz and CKD-EPI equations exhibit poor agreement in AYAs with CKD before and during the transition period. Concordance rises steadily as AYAs age but it is certain that differences between equations remain even after age 18, though these differences were less dramatic for female patients in our study than for male patients. Our findings suggest that switching to the CKD-EPI equation after age 18 may not be an appropriate method for estimating renal function in AYAs with CKD, as the discordance between equations will result in abrupt changes in estimated renal function. Additional studies with measured GFRs, specifically aiming to estimate renal function during the transitioning age period and early adulthood are necessary to understand how to best estimate GFR in AYAs with CKD and track disease progression across the entire life course of this condition.

## Additional files

Additional file 1: Estimated Glomerular Filtration Rate by Each Equation Overall and for Relevant Subgroups. These figures describe trends in eGFR over time based upon the CKD-EPI equation (dashed line) or the Schwartz equation (solid line). Panel A) describes the overall population trajectory, while Panel B) depicts separate trajectories for each third of height Z-score. Panel C) depicts separate trajectories for glomerular and non-glomerular CKD, panel D) shows separate trajectories for males and females, and panel E) shows separate trajectories for African American and non-African American participants. (TIF 618 kb)

Additional file 2: Subject Aggregated Bland-Altman Plots by Age Group. These Bland-Altman plots depict the agreement between our two measurements within age intervals, with each panel from A) to D) representing a separate age group. The closer the central dashed line representing the mean to 0 within each plot, the better the agreement within that age group. Unlike the figure in the main text, each individual can only contribute one dot to the plot collapsed across all creatinine measurements. (TIF 193 kb)

Additional file 3: Subgroup-specific Concordance Correlation Coefficients. This table presents subgroup-specific concordance correlation coefficients beyond those described in the text. (DOCX 13 kb)

## Abbreviations

AYA: Adolescents and young adults
C.C.C.: Concordance correlation coefficient
CKD: Chronic kidney disease
CKD-EPI: Chronic kidney disease epidemiology collaboration
eGFR: Estimated glomerular filtration rate
IDMS: Isotope dilution mass spectrometry
LMM: Linear mixed model
*STAR*_*x*_: Self-management and Transition to Adult-focused healthcare with R_x_ = treatment
