# Supplementary figures and images for: Low agreement between modified-Schwartz and CKD-EPI eGFR in young adults: a retrospective longitudinal cohort study
Source: BMC Nephrol. 2018 Aug 6;19:194. doi: 10.1186/s12882-018-0995-1 (PMC6080537; doi:10.1186/s12882-018-0995-1)

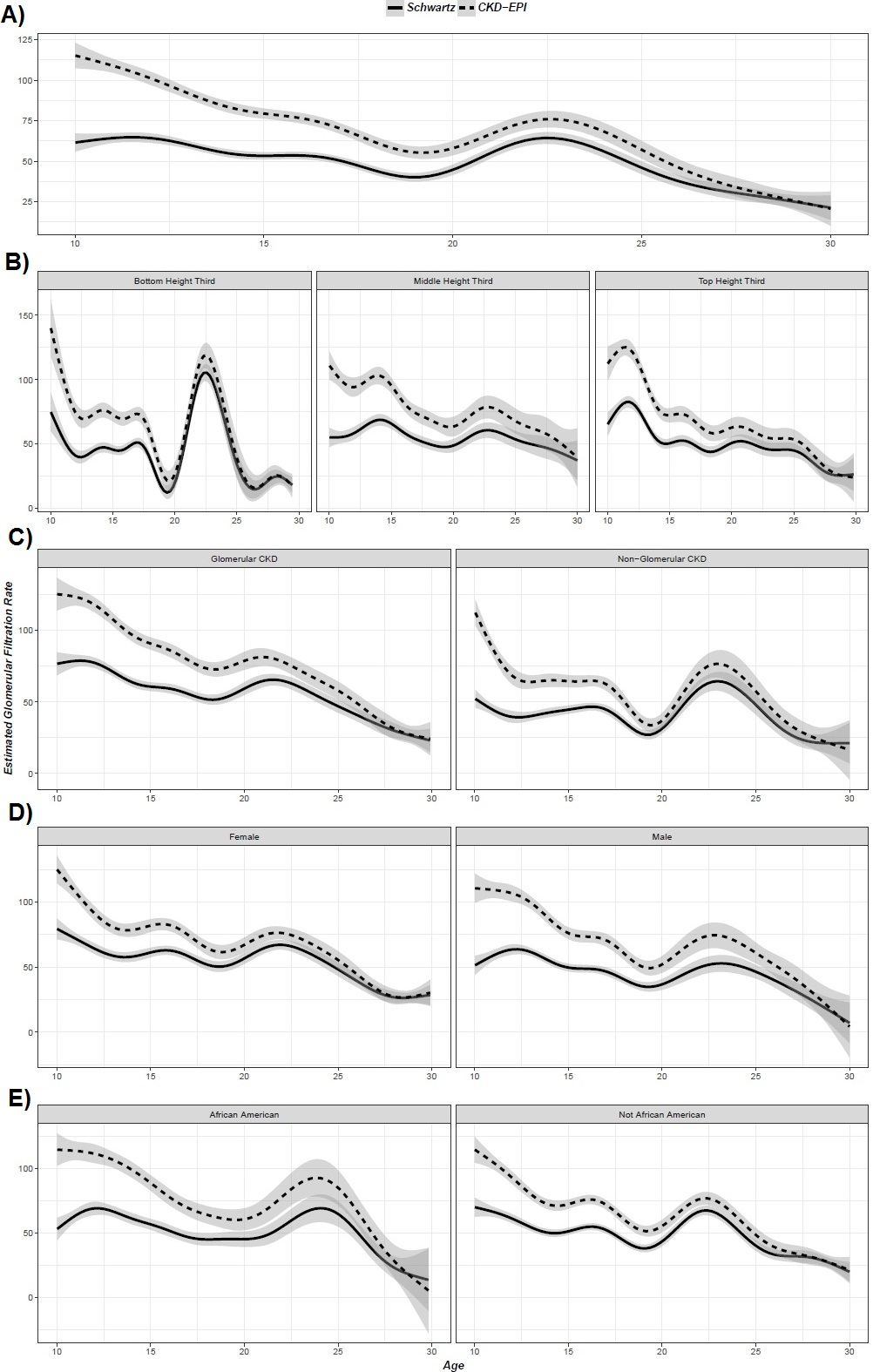

Supplement: Supplementary file 1 — Estimated Glomerular Filtration Rate by Each Equation Overall and for Relevant Subgroups. These figures describe trends in eGFR over time based upon the CKD-EPI equation (dashed line) or the Schwartz equation (solid line). Panel A) describes the overall population trajectory, while Panel B) depicts separate trajectories for each third of height Z-score. Panel C) depicts separate trajectories for glomerular and non-glomerular CKD, panel D) shows separate trajectories for males and females, and panel E) shows separate trajectories for African American and non-African American participants. (TIF 618 kb) [file 12882_2018_995_MOESM1_ESM.tif]

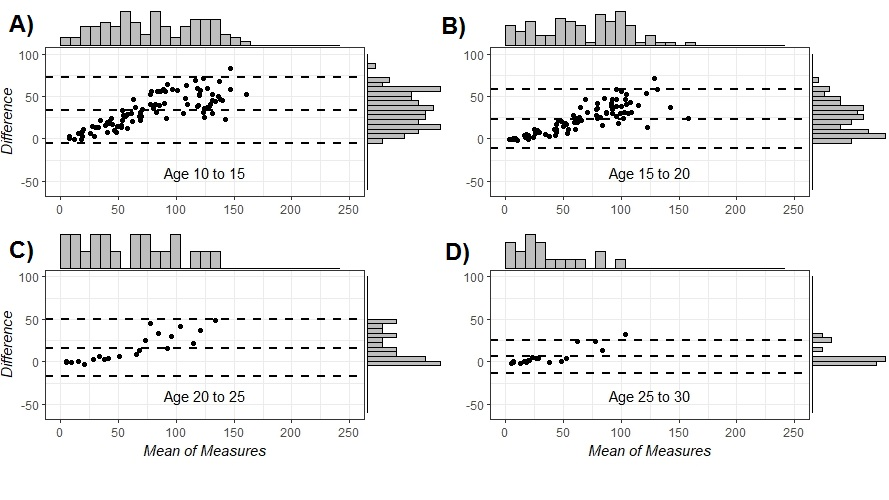

Supplement: Supplementary file 2 — Subject Aggregated Bland-Altman Plots by Age Group. These Bland-Altman plots depict the agreement between our two measurements within age intervals, with each panel from A) to D) representing a separate age group. The closer the central dashed line representing the mean to 0 within each plot, the better the agreement within that age group. Unlike the figure in the main text, each individual can only contribute one dot to the plot collapsed across all creatinine measurements. (TIF 193 kb) [file 12882_2018_995_MOESM2_ESM.tif]
