# Supplementary material for: Low agreement between modified-Schwartz and CKD-EPI eGFR in young adults: a retrospective longitudinal cohort study
Source: BMC Nephrol. 2018 Aug 6;19:194. doi: 10.1186/s12882-018-0995-1 (PMC6080537; doi:10.1186/s12882-018-0995-1)
Supplement: Supplementary file 3 — Subgroup-specific Concordance Correlation Coefficients. This table presents subgroup-specific concordance correlation coefficients beyond those described in the text. (DOCX 13 kb) [file 12882_2018_995_MOESM3_ESM.docx]

| Population | C.C.C.* age 10-15 (95% C.I.) | C.C.C.* age 15-20 (95% C.I.) | C.C.C.* age 20-25 (95% C.I.) | C.C.C.* age 25-30 (95% C.I.) |
| --- | --- | --- | --- | --- |
| Full Cohort | 0.74 (0.67, 0.79) | 0.78 (0.71, 0.84) | 0.80 (0.70, 0.87) | 0.82 (0.70, 0.90) |
| Stratified by gender |  |  |  |  |
| Male | 0.69 (0.59, 0.77) | 0.73 (0.62, 0.82) | 0.76 (0.59, 0.86) | 0.76 (0.52, 0.89) |
| Female | 0.82 (0.73, 0.88) | 0.88 (0.80, 0.93) | 0.88 (0.79, 0.94) | 0.92 (0.83, 0.96) |
| Stratified by race |  |  |  |  |
| Non-African American | 0.79 (0.71, 0.85) | 0.82 (0.74, 0.88) | 0.84 (0.73, 0.91) | 0.90 (0.81, 0.95) |
| African-American | 0.70 (0.59, 0.79) | 0.74 (0.59, 0.84) | 0.77 (0.57, 0.88) | 0.74 (0.41, 0.90) |
| Stratified by Kidney Disease |  |  |  |  |
| Glomerular | 0.72 (0.62, 0.79) | 0.72 (0.62, 0.81) | 0.76 (0.62, 0.85) | 0.83 (0.69, 0.91) |
| Non-Glomerular | 0.74 (0.61, 0.83) | 0.87 (0.77, 0.93) | 0.86 (0.71, 0.94) | 0.83 (0.46, 0.96) |
| Height Z-Score |  |  |  |  |
| Bottom third | 0.72 (0.58, 0.82) | 0.76 (0.56, 0.87) | 0.80 (0.54, 0.92) | 0.94 (0.70, 0.99) |
| Middle third | 0.73 (0.61, 0.81) | 0.79 (0.68, 0.87) | 0.80 (0.66, 0.89) | 0.82 (0.60, 0.93) |
| Top third | 0.74 (0.63, 0.82) | 0.78 (0.65, 0.86) | 0.78 (0.58, 0.89) | 0.83 (0.65, 0.92) |
| *C.C.C: Concordance Correlation Coefficient | | | | |

**Supplementary Table 1: All Population Concordance Correlation Coefficient Results**
